# I Know How You Feel: The Warm-Altruistic Personality Profile and the Empathic Brain

**DOI:** 10.1371/journal.pone.0120639

**Published:** 2015-03-13

**Authors:** Brian W. Haas, Michael Brook, Laura Remillard, Alexandra Ishak, Ian W. Anderson, Megan M. Filkowski

**Affiliations:** 1 Department of Psychology, University of Georgia, Athens, GA, United States of America; 2 Interdisciplinary Neuroscience Graduate Program, University of Georgia, Athens, GA, United States of America; 3 Department of Psychiatry and Behavioral Sciences, Northwestern University Feinberg School of Medicine, Chicago, IL, United States of America; University of Vienna, AUSTRIA

## Abstract

The ability to empathize with other people is a critical component of human social relationships. Empathic processing varies across the human population, however it is currently unclear how personality traits are associated with empathic processing. This study was designed to test the hypothesis that specific personality traits are associated with behavioral and biological indicators of improved empathy. Extraversion and Agreeableness are personality traits designed to measure individual differences in social-cognitive functioning, however each trait-dimension includes elements that represent interpersonal social functioning and elements that do not represent interpersonal social functioning. We tested the prediction that interpersonal elements of Extraversion (Warmth) and Agreeableness (Altruism) are associated with empathy and non-interpersonal elements of Extraversion and Agreeableness are not associated with empathy. We quantified empathic processing behaviorally (empathic accuracy task using video vignettes) and within the brain (fMRI and an emotional perspective taking task) in 50 healthy subjects. Converging evidence shows that highly warm and altruistic people are well skilled in recognizing the emotional states of other people and exhibit greater activity in brain regions important for empathy (temporoparietal junction and medial prefrontal cortex) during emotional perspective taking. A mediation analysis further supported the association between warm-altruistic personality and empathic processing; indicating that one reason why highly warm-altruistic individuals may be skilled empathizers is that they engage the temporoparietal junction and medial prefrontal cortex more. Together, these findings advance the way the behavioral and neural basis of empathy is understood and demonstrates the efficacy of personality scales to measure individual differences in interpersonal social function.

## Introduction

What makes one person better at empathy than another? Empathy is the ability to share or recognize emotions experienced by another person [1,2]. The Big 5 model of personality includes two trait-dimensions often associated with prosociability in general: Extraversion and Agreeableness. However, both Extraversion and Agreeableness contain a diverse set of elements; some elements of Extraversion and Agreeableness represent the tendency to be prosocial while other elements do not [3,4]. This study was designed to test the hypothesis that elements, within Extraversion of Agreeableness, that represent interpersonal social function, are associated with improved empathic accuracy and increased brain activity within the “empathy/theory of mind network.”

The trait-dimensions within the Big 5 model of personality are designed to measure stable cognitive, social and affective tendencies throughout the human lifespan [5,6]. Extraversion is a trait that generally characterizes the tendency towards social motivation, reward sensitivity and excitement seeking [7,8]. Someone scoring high in Extraversion may be extremely close with his or her family and friends (i.e., warm), or may be highly motivated to attend loud concerts (i.e., excitement seeking), or both. In terms of empathic processing, some studies have shown an association between Extraversion and improved empathic processing [9–11], while others have not [12,13]. Agreeableness is a trait that characterizes the tendency to be kind, considerate and cooperative with others [14]. Someone scoring high in Agreeableness may be motivated to help others in need (i.e., altruistic), or may tend to be extremely up front with other people (i.e., straightforward), or both. In terms of empathic processing, some studies have shown an association between Agreeableness and improved empathic processing [15,16], while others have not [17,18]. This mixed pattern of results suggests that there may be some elements of Extraversion the Agreeableness that correspond to empathic processing, while some elements do not.

Several models of personality have characterized an array of elements that underlie the Big 5 trait-dimensions [3,4,19–22]. Within the NEO Personality Inventory, Warmth (E1: Extraversion) and Altruism (A3: Agreeableness) are two facets designed to measure prosociability and interpersonal social function [23]. Warmth is a facet of Extraversion that represents the tendency to be interpersonally intimate with others. Those scoring high on Warmth tend to be highly affectionate and easily form close attachments with other people [23]. Some behavioral research shows an association between the Warmth facet of Extraversion and empathic processing [24]. Additionally, brain imaging research shows that trait warmth (measured by the Interpersonal Adjective Scale) is associated with the structure of brain regions important for empathy [25]. Sollberger et al., [25] showed that higher trait warmth is associated with greater gray matter volume within the medial prefrontal cortex (PFC). These findings suggest that warmth may be an element of Extraversion that represents interpersonal closeness and improved empathic processing.

Altruism is a facet of Agreeableness that represents the tendency towards selflessness, interpersonal motivation and concern for others [22]. The Altruism facet is conceptualized to represent a similar personality profile as Social Interest, initially described by Alder and colleagues [26]. People scoring high on the Altruism facet of Agreeableness tend to be invested in interpersonal relationships [27] and are evaluated as high in nurturance and affiliation by others [28]. Behavioral research indicates a link between altruism and empathic processing [29,30] which are in support of theoretical models such as the empathy-altruism hypothesis [31]. Some brain imaging research shows that behavioral measures of altruism are associated with the function of brain regions important for empathy and theory of mind [32]. Specifically, Morishima et al., [32] showed that higher behavioral measures of altruism are associated with greater temporoparietal activity (TPJ). The reason why some studies have failed to find an association between personality (Extraversion and Agreeableness) and empathic processing may be because both interpersonal and non-interpersonal elements were combined to characterize personality [12,13,17,18]. Because Warmth (E1: Extraversion) and Altruism (A3: Agreeableness) measure prosociability and interpersonal functioning specifically, the use of these facets may improve the way personality scales can be used to quantify the tendency to be empathic.

In this study, we sought to test the prediction that individuals characterized as warm and altruistic exhibit an improved ability to accurately identify the emotional feeling states of other people by using a recently developed, ecologically valid, metric of empathic accuracy [33]. Next, we used functional neuroimaging to test if individuals characterized as warm and altruistic exhibit increased brain activity within the empathy/theory of mind network [34,35], during emotional perspective taking. Within the brain, we focused our analyses on the TPJ, medial PFC, precuneus, and anterior insula [34,35]. Lastly, we performed a mediation analysis designed to investigate how brain activity within the empathy/theory of mind network mediates the association between the warm-altruistic personality profile and empathic accuracy.

## Materials and Methods

### Participants

We recruited 50, fluent English-speaking (31 females, 19 males; mean age = 20.14 years, SD = 1.95 years, 48 right-handed) adults from the University of Georgia and surrounding community to participate in behavioral testing and neuroimaging. All participants were screened for neurological conditions and MRI counter indications. All participants provided written informed consent as detailed in the Declaration of Helsinki, and the University of Georgia Institutional Review Board approved all procedures within this study.

### Procedure

Each participant completed behavioral testing (personality assessment and empathic accuracy task) in a laboratory within the Psychology Department at the University of Georgia. On a separate day, each participant completed an emotional perspective taking task while undergoing fMRI at the University of Georgia Bio-Imaging Research Center (birc.uga.edu).

### Altruistic warm personality profile

Each participant completed the NEO Personality Inventory-3 (NEO PI-3) [36]. The NEO PI-3 covers each of the Big 5 personality trait-dimensions (neuroticism, extraversion, openness, agreeableness, and conscientiousness) and facets for each trait that include E1: Warmth for Extraversion and A3: Altruism for Agreeableness. Personality data were scored to represent T-values, with the population mean defined as T = 50 and one standard deviation of T = 10. The sample scores for Extraversion (M = 53.45, SD = 11.81), E1: Warmth (M = 50.45, SD = 10.79), Agreeableness (M = 46.90, SD = 11.33) and A3: Altruism (M = 49.86, SD = 9.61) were well within the range of the normal non-clinical population. Extraversion and Agreeableness were not significantly correlated with one another (*r* = -.13, *p* = .38), while the E1: Warmth and A3: Altruism facet scores were significantly correlated (*r* = .42, *p* <. 01).

In order to characterize individual differences in the warmth-altruism personality profile, composite scores were calculated by averaging T-values across the Warmth and Altruism facets. Thus, higher warmth-altruism composite scores represent being high on both the Warmth and Altruism facets. An examination of the internal consistency for all items included with the within Warmth and Altruism facets combined showed a Cronbach’s alpha of. 77.

### Empathic accuracy task

Each participant completed an empathic accuracy task, as detailed in Brook & Kooson [33]. The design of this task is based on the methodological approach initially proposed by Ickes [37]. In this task, participants (perceivers) view a series of short video clips of adult volunteers (targets) relaying past emotional experiences. Targets within the video clips were not actors. Each target was a volunteer and was asked to recount an actual emotional experience that they had. After each video clip was obtained, each target completed the tripartite hierarchy of emotion words [38] to describe how they felt when they were relating their story.

Following each video clip, each participant (perceiver) was also instructed to complete the tripartite hierarchy of emotion words [38] to describe the emotions they believed the target experienced while recounting their emotional experience, during the video clip. The primary level of the hierarchy consists of six basic emotions (love, joy, surprise, anger, fear and sadness). The secondary level consists of 25 more complex descriptors of emotional states (e.g., affection, zest, irritation, nervousness and disappointment). The secondary level is nested within each basic emotion. Lastly, the tertiary level is comprised of descriptors of complex mental states (e.g., longing, contentment, resentment, embarrassment) nested within each secondary emotion. Following each video, participants were asked to identify and rank-order (from most to least) the emotions they believed the target in the video felt at that moment as they were recounting their story. Potential responses consisted of the 25 secondary emotions from the hierarchy described above. Each secondary emotion was accompanied by a parenthesized list of its corresponding tertiary emotions to provide additional emotional context. Targets and subjects were free to endorse as few or as many (i.e., all 25) emotions as they believed were accurate, however there were no targets or perceivers that endorsed all 25 emotions on any trial.

Empathic accuracy was operationalized as the degree of correspondence (0–2 Likert scale) between a perceiver’s rating of the most salient emotion (i.e., the emotion ranked #1) and each target’s rating of the most salient emotion. Specifically, a score of 2 was assigned if the highest ranked secondary emotion by the participant (perceiver) was also the highest ranked secondary emotion endorsed by the target. A score of 1 was assigned if the highest ranked secondary emotion by the participant (perceiver) was within the response set endorsed by the target. A score of 0 was assigned if the highest ranked secondary emotion by the participant (perceiver) was not endorsed by the target. Empathic accuracy scores, for each subject, were calculated based on the sum of scores across all trials (n = 13). This task was designed to enhance the ecological validity of empathic accuracy measurement across diverse populations. Psychometric analysis of data obtained using this task shows high construct validity with respect to other measures of empathic processing [33].

### Emotional perspective taking task

**Fig 1:**
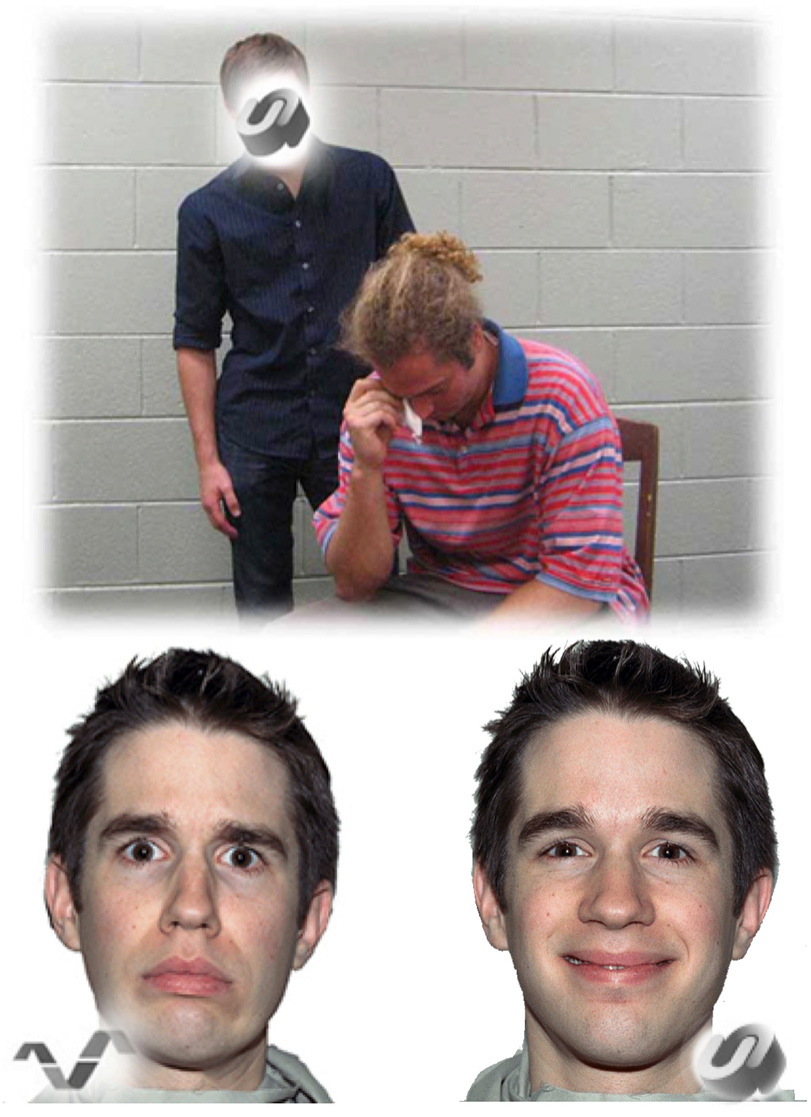
Examples of stimuli used for the emotional perspective taking task performed during functional neuroimaging. Participants were asked to make two types of decisions. During emotional perspective taking, participants were instructed to take into account the social interaction within a scene presented on the top of the screen, and to decide which of two emotional facial expressions best matches the face. During shape matching (control condition), participants were instructed to match the shape imbedded within the social scene with one of two shapes presented on the bottom of the screen. The individuals in this image have given written informed consent to publish this image.

Each participant underwent fMRI while completing an emotional perspective taking task (Fig. 1; The individuals in the figure have given written informed consent to publish this image). The conceptual design of this task is based on the paradigm developed by Derntl et al., [39]. Prior neuroimaging research demonstrates that this task reliably engages brain activity within the empathy/theory of mind network [39].

In this task, participants are instructed to make two types of decisions. For emotional perspective taking, each participant is instructed to take into account the social interaction within a scene presented on the top of the screen, and to decide which of two emotional facial expressions best matches the face that is “blanked out” within the social scene. As a control condition, each participant is instructed to match the shape embedded within the social scene with one of two shapes presented on the bottom of the screen. Thus, the visual characteristics are identical across conditions and accuracy in both conditions is contingent upon visually probing the top and bottom of the screen/stimuli. Pilot testing demonstrated that accuracy is higher during emotion perspective taking than shape matching, and reaction time latencies are longer during emotion perspective taking than shape matching.

Social scene stimuli were created by taking photographs of volunteers while displaying one of five types of social-emotional interactions: angry, fear, sad, neutral, happy. Emotional facial expression stimuli were obtained from a standardized database (NimStim Face Stimulus Set) [40]. A total of 14 trials for each category (angry, fear, sad, neutral, happy) were created. Pilot testing showed that each social-emotional interaction scene was correctly labeled to each emotion category (>95%).

Each trial was presented for a total of 4 seconds and was preceded by a letter fixation-cue (“E” for match emotions or “S” for match shapes). Trials were presented pseudo-randomly in blocks of 3 or 4 trials in length. The total duration of the experiment was 7 minutes and 12 seconds. Each participant responded to a total of 70 trials (35: emotion perspective taking, 35: match shapes). The number of trials for each category (angry, fear, sad, neutral, happy) was equal within the emotion perspective taking and shape matching control conditions. Immediately prior to entering the MRI scanner, each participant completed a practice version of the task. The practice version did not include any stimuli included in the version of the task used during fMRI data collection.

### Image acquisition

Whole-brain imaging data were acquired on a GE-Signa 3.0T scanner (General Electric, Milwaukee, WI) at the University of Georgia Bio-Imaging Research Center (birc.uga.edu). A total of 213 functional images were acquired using a gradient echo T2*-weighted echoplanar imaging (EPI) scan and were obtained using a flip angle of 90°, repetition time (TR) = 2.0s, echo time (TE) = 25ms, 40 slices, and a field of view (FOV) = 220mm x 220mm, matrix = 64 x 64. For structural whole brain images, a three-dimensional high-resolution spoiled gradient scan (FSPGR) (repetition time, 24 msec; echo time, 4.5 msec; flip angle, 20°; matrix size, 256 x 256; field of view, 25.6 cm; slice thickness, 1.0 mm; 164 contiguous slices) was conducted.

### fMRI Preprocessing

Functional data were preprocessed and statistically analyzed using SPM8 software (Wellcome Department of Imaging Neuroscience, London, UK) and implemented through Matlab R2012a (www.mathworks.com). The images were temporally realigned to the middle slice, spatially realigned to the first in the time series. The images were then coregistered and spatially normalized into standard stereotactic space (MNI template) and spatially smoothed with an 8mm full width-half maximum isotropic Gaussian filter.

### Hypothesis testing

A regression analysis within SPSS software (IBM Corp., Armonk, NY) was used to test the significance of the association between warm-altruistic composite scores and empathic accuracy using a *p* <. 05 (two-tailed) significance threshold. Next, we used a regression analysis within SPM8 software to test the significance of the association between warm-altruistic composite scores and brain activity during emotion perspective taking. Lastly, we used the SPSS macro by Preacher and Hayes [41] to perform a mediation analysis to test the significance of brain activity within the empathy/theory of mind network mediating the association between warm-altruistic composite scores and empathic accuracy.

### Statistical analysis: fMRI

Statistical analyses of fMRI data were initiated by comparing BOLD signal acquired during the emotion attribution to shape matching conditions (emotion perspective taking > match shapes conditions). Each block, within each condition, was modeled based on a convolution to the hemodynamic response function and represented BOLD signal acquired throughout each block (boxcar), including fixation-cues. Data within each model were high pass filtered using default settings (128s). On a group level, *t*-contrast maps were entered into a random effects model. Lastly, a whole-brain regression analysis was conducted, with warm-altruistic composite scores entered as the independent variable and contrast estimates (emotion perspective taking > match shapes) entered as the dependent variable. Unless otherwise specified, neuroimaging results are reported at a threshold of *p* <. 005, voxel extent of *k* = 60, two-tailed and uncorrected, to preserve the balance between sensitivity and false positive rates [42].

### Mediation analysis

Mediation analyses, in the context of neuroimaging, can provide evidence that neural activity accounts for the covariation between two behavioral variables. We performed a mediation analysis to explore how neural activity within the empathy/theory of mind network mediates the association between the warm-altruistic personality profile and empathic accuracy. The mediation model included two behavioral variables: warm-altruistic composite scores and empathic accuracy scores; and neural activity variables (contrast estimates for emotional perspective taking > match shapes) extracted from two regions of interest (TPJ and medial PFC). Signal from the TPJ and medial PFC were extracted using a conjunction analysis. Specifically we conducted two separate whole-brain regressions, with one regression for warmth-altruism as the independent variable and the other with empathic accuracy as the independent variable. Thus voxels were located that were significantly associated with both warm-altruistic composite scores and empathic accuracy scores. Next, contrast estimates (emotion matching > shape matching) were extracted for each subject and subsequently entered into a mediation model within SPSS. The significance of the overall mediation model and the independence of indirect effects in multiple mediation analysis was obtained using a 95% confidence interval with 5000 bootstrap resamples as implemented by the SPSS macro by Preacher and Hayes [41]. All of the variables (1. warm-altruistic composite score, 2. medPFC contrast estimate, 3. TPJ contrast estimate and 4. empathic accuracy) were entered simultaneously to calculate the significance of the overall model as well as the significance of each path.

## Results

### Warm-altruistic personality profile and empathic accuracy

Regression analysis, with warm-altruistic composite scores entered as the independent variable, and empathic accuracy scores entered as the dependent variable, showed a statistically significant association. Higher warm-altruistic composite scores were associated with improved empathic accuracy, *r* = .35, *F*(1,48) = 6.61, *p* = .013. Spearman’s rank correlation coefficient for the association between warm-altruistic composite scores and empathic accuracy was also statistically significant (rho = .303, p = .033). The association remained significant when sex was entered as a covariate (*p* = .007). The E1: Warmth and A3: Altruism facet scores, independently, each exhibited a significant association with improved empathic accuracy (Warmth: *r* = .32, *p* < 05; Altruism: *r* = .26, *p* <. 05). We also performed an analysis with domain scores for Extraversion and Agreeableness each entered as independent variables and empathic accuracy entered as the dependent variable. The results indicated that Extraversion domain scores were not associated with empathic accuracy (*r* = .15, *p* = .30), and Agreeableness domain scores were associated with empathic accuracy (*r* = .33, *p* = .02). Lastly, we examined the association between a subset of Extraversion and Agreeableness facets not designed to characterize interpersonal social functioning (E3: Assertiveness, E5: Excitement-seeking, A4: Straightforwardness, and A5: Modesty) and empathic accuracy. The results of this analysis indicated that the Assertiveness, Excitement-seeking, Straightforwardness and Modesty facets were not associated with empathic accuracy (E3: *p* = .88, E5: *p* = .19, A4: *p* = .17, A5: *p* = .23).

### Emotional perspective taking task behavioral performance

Accuracy and reaction time (RT) data were collected for emotional perspective taking task (completed with undergoing fMRI). The overall accuracy was 92.9% (89% for emotional perspective taking and 97% for shape matching). The average RT and standard deviation was 2.20±.25s for emotional perspective taking and 1.31±.21s for shape matching. There were no statistically significant associations between behavioral measures during the emotional perspective taking task (accuracy or RT) and empathic accuracy scores (video task) or personality measures (Warmth, Altruism or Warm-altruistic composite scores).

### Altruistic-Warmth personality profile and brain activity

**Fig 2:**
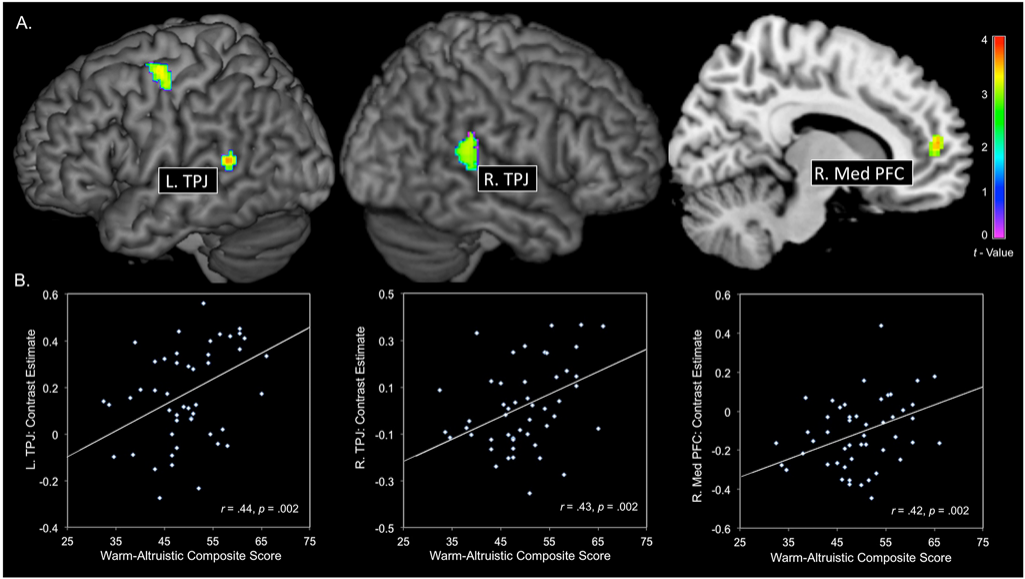
A. Results of whole brain analysis, within warm-altruistic composite scores predicting brain activity during emotional perspective taking > shape matching. A total of four statistically significant clusters are reported (bilateral TPJ, right medial PFC, and left premotor cortex). **B**. Data from each cluster within the empathy/theory of mind network are extracted (*y*-axis) and plotted against altruism-warmth composite scores (*x*-axis). TPJ: temporoparietal junction; PFC: prefrontal cortex; R: right; L: Left.

The results of whole brain analysis for emotional emotion perspective taking > match shapes (with no covariates) are shown within the Supplementary Results section. The results of the whole brain analysis, with warm-altruistic composite scores entered as the independent variable and contrast estimates (emotion perspective taking > match shapes) entered as the dependent variable, revealed a total of 4 statistically significant clusters within the bilateral TPJ (right: MNI: -58, -46, 14; *t* = 3.56, *p* <. 001, k = 67 voxels; left MNI: 60, -36, 22; *t* = 3.93, *p* = .002, k = 116 voxels), right rostral medial PFC (MNI: 10, 52, 12; *t* = 3.50, *p* <. 001, k = 74 voxels) and the left premotor cortex (MNI: -44, -8, 62; t = 3.31, p = .001, k = 116 voxels) (Fig. 2A). We carried out a series of steps to confirm the anatomical location for each TPJ cluster. We generated structural and functional connectivity based ROIs, as reported by Mars et al [43]. For each cluster, we quantified the number of voxels, correlated with warm-altruism, within the anterior TPJ (14mm radius sphere; MNI ± 58, -37, 20), posterior TPJ (14mm radius sphere; MNI ± 54, -55, 20) and the inferior parietal lobule (14mm radius sphere; MNI ± 49, -46, 46). The results of this analysis showed that for the left TPJ cluster (67 voxels), 43 voxels fell within the anterior TPJ, 20 voxels fell within the posterior TPJ, and 0 voxels fell within the inferior parietal lobule. For the right TPJ cluster (116 voxels), we found that 113 voxels fell within the anterior TPJ, 0 voxels fell within the posterior TPJ, and 0 voxels fell within the inferior parietal lobule.

To further investigate the association between the warm-altruistic personality profile and activity within the empathy/theory of mind network, we extracted mean contrast estimates from each cluster located within the left and right TPJ and medial PFC (Fig. 2B). For each cluster, we calculated regression coefficients and examined the strength of each correlation when sex and handedness were entered as covariates. This analysis demonstrated that the association between warm-altruistic composite scores and activity within the left and right TPJ and medial PFC remained statistically significant after accounting for sex and handedness.

Next, we tested for associations between each personality facet independently and contrast estimates within each cluster. This analysis demonstrated that Warmth was associated with activity within the TPJ (L. TPJ: *r* = .36; R. TPJ: *r* = .34) but was not significantly associated with activity within the Med PFC (*r* = .25, *p* = .06) and Altruism was associated with activity in all clusters (L. TPJ: *r* = .37; R. TPJ: *r* = .39; Med PFC: *r* = .45). Lastly, we examined the association between a subset of Extraversion and Agreeableness facets not conceptualized to characterize interpersonal social functioning (E3: Assertiveness, E5: Excitement-seeking, A4: Straightforwardness, and A5: Modesty) and brain activity during emotional perspective taking. The results of this analysis indicated that the Assertiveness, Excitement-seeking, Straightforwardness and Modesty facets were not associated with activity in either the left or right TPJ or the medial PFC.

### Mediation analysis

We performed an exploratory mediation analysis designed to examine if activity within the empathy/theory of mind network mediates the association between the warm-altruistic personality profile and empathic accuracy. The mediation model included warm-altruistic composite scores, contrast estimates within the TPJ and medial PFC (TPJ: MNI: 62, -34, 28; and medial PFC: MNI: -8, 58, 6) and empathic accuracy. Each mediation variable (TPJ and medial PFC contrast estimates) was located and extracted as a result of a conjunction analysis for warm-altruistic composite scores and empathic accuracy scores within each region of interest (TPJ and medial PFC). We performed two separate analyses; initially a whole brain mask was created using the clusters of activation demonstrated to be significantly associated with warm-altruistic composite scores, next this mask was used to locate voxels significantly associated with empathic accuracy scores. Thus, this analysis was performed to identify brain regions correlated with both warm-altruistic composite scores and empathic accuracy (see Discussion section for limitations associated with this method).

**Fig 3:**
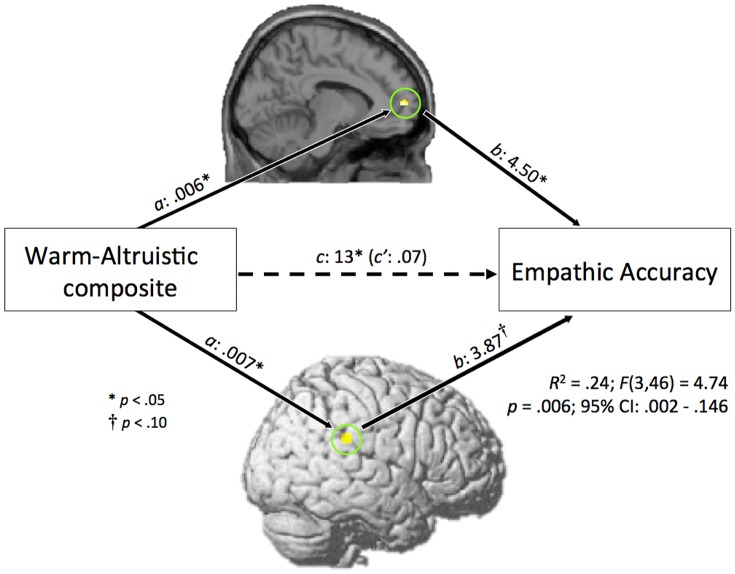
Overall model for mediation analysis. The association between warm- altruistic composite scores and empathic accuracy is mediated by activity within the medial prefrontal cortex (PFC) and temporoparietal junction (TPJ).

The overall mediation model is presented in Fig. 3. The direct path between warm-altruistic composite scores and empathic accuracy (*c* path: no mediator variables entered) was statistically significant (*B* = .13, *t*(48) = 2.58, *p* = .013). However, when the TPJ and medial PFC mediator variables were entered, the association between warm-altruistic composite scores and empathic accuracy (*c*’ path) no longer reached statistical significance (*B* = .07, *t*(48) = 1.37, *p* = .18). Both *a* paths (*a*_1_: warm-altruistic to medial PFC, and a_2_: warm-altruistic to TPJ) reached statistical significance (*a*_1_: *B* = .006, *t*(48) = 2.06, *p* = .04; a_2_: *B* = .007., *t*(48) = 2.15, *p* = .03). For *b* paths (*b*_1_: medial PFC to empathic accuracy, and *b*_2_: TPJ to empathic accuracy), the association between medial PFC activity and empathic accuracy was statistically significant (*b*_1_: *B* = 4.50, *t*(48) = 2.06, *p* <. 05), while the association between TPJ activity and empathic accuracy approached statistical significance (*b*_2_: *B* = 3.87, *t*(48) = 1.87, *p* = .068). Lastly, results indicated the mediating role of the medial PFC and TPJ in the association between warm-altruistic composite scores and empathic accuracy (*B* = .058; CI = .002 to. 146). The estimates of the indirect effects through the TPJ and medial PFC separately were as follows: TPJ; *B* = .027, CI = -.003 - .105; Medial PFC: *B* = .031, CI = .0003 - .096. The overall mediation model remained statistically significant when sex and handedness were entered as covariates (*p* <. 01).

## Discussion

In this study, we demonstrate that people characterized as highly warm and altruistic exhibit an improved ability to recognize the emotional feeling states of other people. Furthermore, highly warm and altruistic people exhibit increased neural activity in key brain regions involved in empathy and theory of mind during emotional perspective taking. Lastly, activity within the medial PFC and TPJ mediates the association between the warm-altruistic personality profile and empathic accuracy, suggesting that one reason why highly warm and altruistic people are empathically accurate is that they engage the empathy/theory of mind network more. These findings further elucidate the way specific elements of Extraversion and Agreeableness are theorized to reflect tendencies involved in interpersonal social function [3,4,21,22] and more specifically a disposition towards being empathic.

The findings of this study advance the way underlying elements of the Big 5 trait-dimensions are understood. Empirical research shows that there exists intermediary levels between traits and facets and that both traits and facets have distinct biological substrates [20,44,45]. DeYoung, et al., [20] performed a factor analysis to reveal a level of personality that exists between facets and traits. At this level, DeYoung et al., [20] showed that Extraversion can be divided into Enthusiasm versus Assertiveness and Agreeableness can be divided into Compassion versus Politeness. Enthusiasm is linked to the rewarding nature of social affiliation, whereas Compassion reflects affiliation driven by concern or empathy. Within this model, the Warmth facet, within the NEO, loads onto the Enthusiasm construct of Extraversion, while Altruism loads onto Compassion and Politeness constructs of Agreeableness. The current findings are in support of this structure and also indicate that there are sets of characteristics that Warmth and Altruism share. Specifically our findings indicate that Warmth and Altruism consist of overlapping behavioral and biological factors related to interpersonal social functioning and the tendency to be empathic.

We measured empathic accuracy using a task consisting of short video clips. The use of video clips of real emotional events, relayed by non-actor volunteers, including sound and biological motion provides a highly naturalistic and ecologically valid metric of empathic accuracy [37]. Several theories of empathic processing deconstruct empathy into aspects that include (but are not limited to) emotion contagion, perception-action coupling, and theory of mind/cognitive empathy [2,35,46]. The task used in our study measures the ability to recognize the emotional states of other people. Prior research using this task [33] demonstrates an association between empathic accuracy and self-report measures of perspective-taking (PT) [47], conceptualized as a metric of cognitive empathy. These findings suggest that the warm-altruistic personality profile is associated with cognitive empathy and may not be associated with other forms of empathy. This however, was not directly compared in this study. Thus further research is required to support this hypothesis.

The results of the fMRI analysis demonstrate that the warm-altruistic personality profile is associated with greater TPJ and medial PFC activity during emotional perspective taking. These findings are consistent with evidence that TPJ and medial PFC are important for both empathy and theory of mind [34,35]. The TPJ is strongly implicated in theory of mind and perspective taking. Temporary lesions (as with TMS) of the TPJ reduce a person’s ability to complete false belief tasks and attribute mental states to other people [48,49]. These findings suggest that highly warm and altruistic people may be better able to put themselves in other people’s perspectives. The medial PFC is strongly implicated in processing the self and emotional empathy [50]. Medial PFC activity is often observed when people think about self-relevant information [51,52] and during social decision making [53]. Together with the current findings, this indicates that highly warm and altruistic people are better able to incorporate their own values and emotional states during social decision making. We did not observe, however, that the altruistic-warm personality profile was associated with activity in other brain regions commonly activated during empathy and theory of mind tasks, such as the precuneus and anterior insula. The fact that we did not observe activity in other brain regions within the empathy/theory of mind network may indicate that there are some aspects of empathy that do not vary according to warmth-altruism. Other personality dimensions may be better suited to capture variance in neural activity within other brain regions within the empathy/theory of mind network.

We also observed that higher warm-altruistic composite scores were associated with greater right premotor cortex activity. This finding was unexpected, however this finding may in part represent how the premotor cortex functions to simulate the actions of other people (e.g. motor imagery) [54]. For example, Grèzes and Decety [55] found that premotor cortex activity increases when participants mentally simulate a motor movement. The social scene stimuli used in the current task, emotional perspective taking, includes gestures that imply movement and action. Highly warm and altruistic people may exhibit greater premotor activity during this task because they are mentally simulating the motor gestures displayed within the social scenes.

We used an exploratory mediation analysis to test if TPJ and medial PFC activity mediates the association between the warm-altruistic personality profile and empathic accuracy. The results of this exploratory analysis suggest that one reason why highly warm and altruistic people exhibit improved empathic accuracy is that they engage the TPJ and medial PFC more strongly during emotional perspective taking. The results of the whole brain analysis with warm-altruistic composite scores revealed a total of 3 clusters within the empathy/theory of mind network (Fig. 2): bilateral TPJ and medial PFC. However, the mediation model only included activity from 2 regions: the right TPJ and medial PFC (Fig. 3). We found that activity within the right TPJ mediated the association between the warm-altruistic personality profile and empathic accuracy, while activity within the left TPJ did not. This finding suggests that while highly warm and altruistic people exhibit greater bilateral TPJ activity, the activity within the right TPJ may have a stronger role in terms of improving empathic accuracy. This finding and interpretation is consistent with prior evidence highlighting the specificity of the right TPJ during theory of mind tasks [56]. A limitation of the mediation analysis is that the fMRI data and behavioral data were collected at different time points. This leaves open the possibility that activation observed using fMRI was not specifically related to performance on the empathic accuracy task. Thus the results of the mediation analysis should be considered with caution.

We collected data from a sample of 50 participants, consisting of a larger proportion of females (n = 31) than males (n = 19). We confirmed that the association between personality and dependent variables (empathic accuracy or brain activity) remained statistically significant when sex was entered as a covariate. There is empirical evidence showing that empathy is processed differently according to sex [57]. However, we did not specific test for sex x personality interactions on empathic processing in this study.

There are several other important limitations and caveats of this study that warrant consideration. We used a combined personality, behavioral and neuroimaging approach to test a model linking warmth and altruism to empathy. This model and approach however are limited in terms of understanding people’s motivation and ability to help others. For example, if highly warm and altruistic people are well suited to recognizing that other people are in need, are they also well suited to offer the appropriate type of support? It is also not clear from our results if highly warm and altruistic people are more motivated and/or skilled at empathic processing. Increased brain activity within the medial PFC and TPJ may represent more effort/motivation or a more “highly developed” system. Furthermore, this study is limited in terms of informing developmental models of empathy [58]. Do highly warm and altruistic people exhibit greater activity with the empathy/theory of mind network because they have engaged in empathy more often or are they naturally driven towards empathic processing? Clearly, future research is required to investigate these open questions.

In conclusion, this study provides a link between interpersonal social elements of Extraversion and Agreeableness and empathic processing. Highly warm and altruistic people are more accurate in recognizing emotional states of other people and exhibit increased neural activity in brain regions important for empathy, theory of mind and social cognition.

## Supporting Information

S1 Fig Whole brain results for emotional perspective taking versus shape matching. Areas of significant activation are overlaid on a standardized template of the brain. The upper right panel is a sagittal slice. The lower panel shows activation on axial slices.
(TIF)

S1 Text (DOC)
