# Supplementary figures and images for: I Know How You Feel: The Warm-Altruistic Personality Profile and the Empathic Brain
Source: PLoS One. 2015 Mar 13;10(3):e0120639. doi: 10.1371/journal.pone.0120639 (PMC4359130; doi:10.1371/journal.pone.0120639)

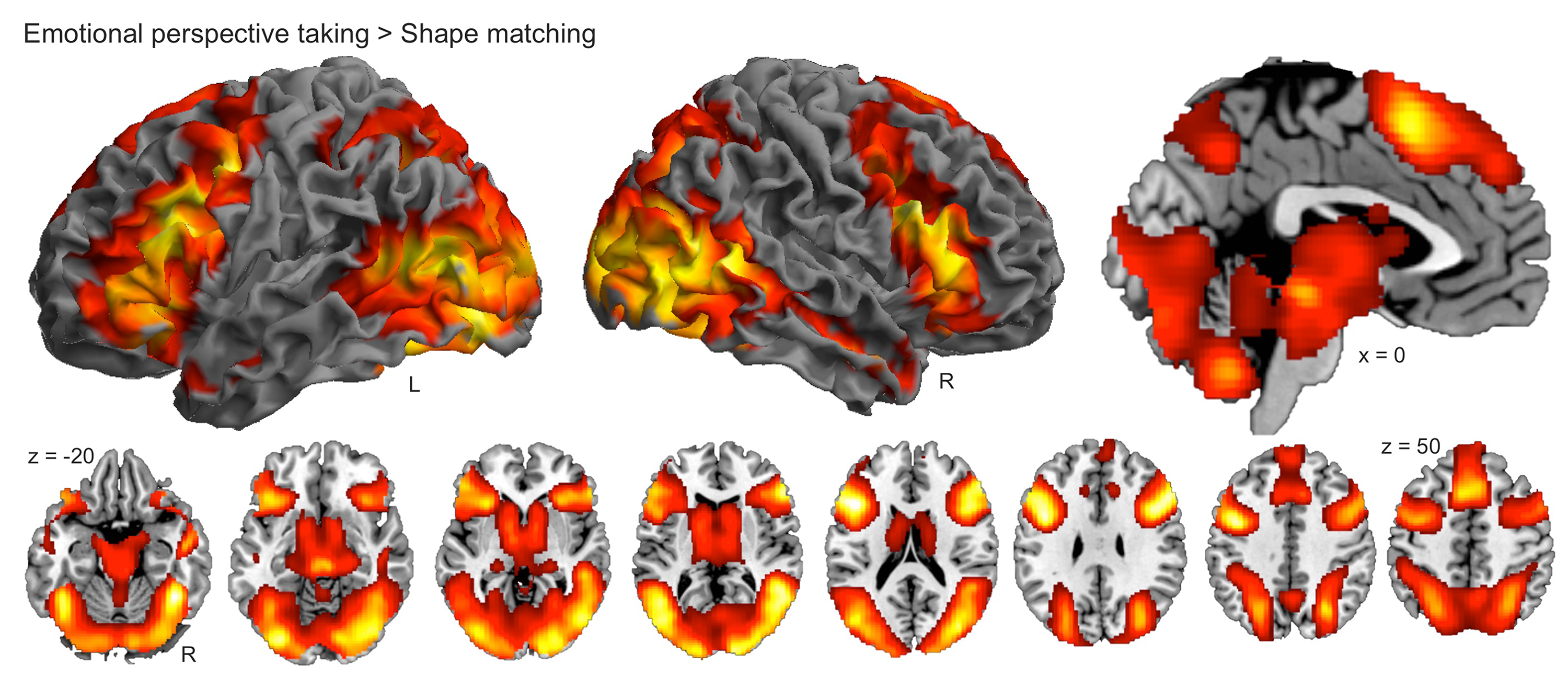

Supplement: S1 Fig — Areas of significant activation are overlaid on a standardized template of the brain. The upper right panel is a sagittal slice. The lower panel shows activation on axial slices. (TIF) [file pone.0120639.s001.tif]
