# Supplementary material for: I Know How You Feel: The Warm-Altruistic Personality Profile and the Empathic Brain
Source: PLoS One. 2015 Mar 13;10(3):e0120639. doi: 10.1371/journal.pone.0120639 (PMC4359130; doi:10.1371/journal.pone.0120639)
Supplement: S1 Text — (DOC) [file pone.0120639.s002.doc]

**Supporting Information**

We performed a whole brain analysis, comparing BOLD signal between the emotion matching and shape matching conditions. Results are presented in **Figure S1**. ROI analysis within areas comprising the empathy/theory of mind network [1,2], demonstrated that emotion matching was associated with greater bilateral anterior TPJ (left: 86 voxels; MNI: -54, -48, 14; *t* = 7.79, *p* < .001; right: 321 voxels; MNI: 52, -48, 16; *t* = 14.11, *p* < .001) posterior TPJ (left: 456 voxels; MNI: -46, -56, 18; *t* = 10.81, *p* < .001; right: 563 voxels; MNI: 50, -60, 14; *t* = 15.35; *p* < .001), medPFC (1257 voxels; MNI: -4, 16, 52; *t* = 12.56; *p* < .001), precuneus (3878 voxels; MNI: 12, -66, 40; *t* = 11.78, *p* < .001) and bilateral insula (left: 407 voxels; MNI: -28, 20, -2; *t* = 11.28, *p* < .001; right: 202 voxels; MNI: 32, 24, 2; *t* = 9.91, *p* < .001) activation compared to the shape matching condition.

**Supporting References:**

1. Van Overwalle F, Baetens K (2009) Understanding others' actions and goals by mirror and mentalizing systems: a meta-analysis. Neuroimage 48: 564-584.

2. Decety J (2011) Dissecting the neural mechanisms mediating empathy. Emotion Review 3: 92-108.
